# Supplementary material for: The interactome of intact mitochondria by cross-linking mass spectrometry provides evidence for coexisting respiratory supercomplexes
Source: Mol Cell Proteomics. 2017 Dec 8;17(2):216–32. doi: 10.1074/mcp.RA117.000470 (PMC5795388; doi:10.1074/mcp.RA117.000470)
Supplement: Supplemental Data [file supp_RA117.000470_133922_0_supp_23978_fzffwf.zip › spectra_annotation/mito_spectra_annotation/249-1-20-1-6-1.pdf]

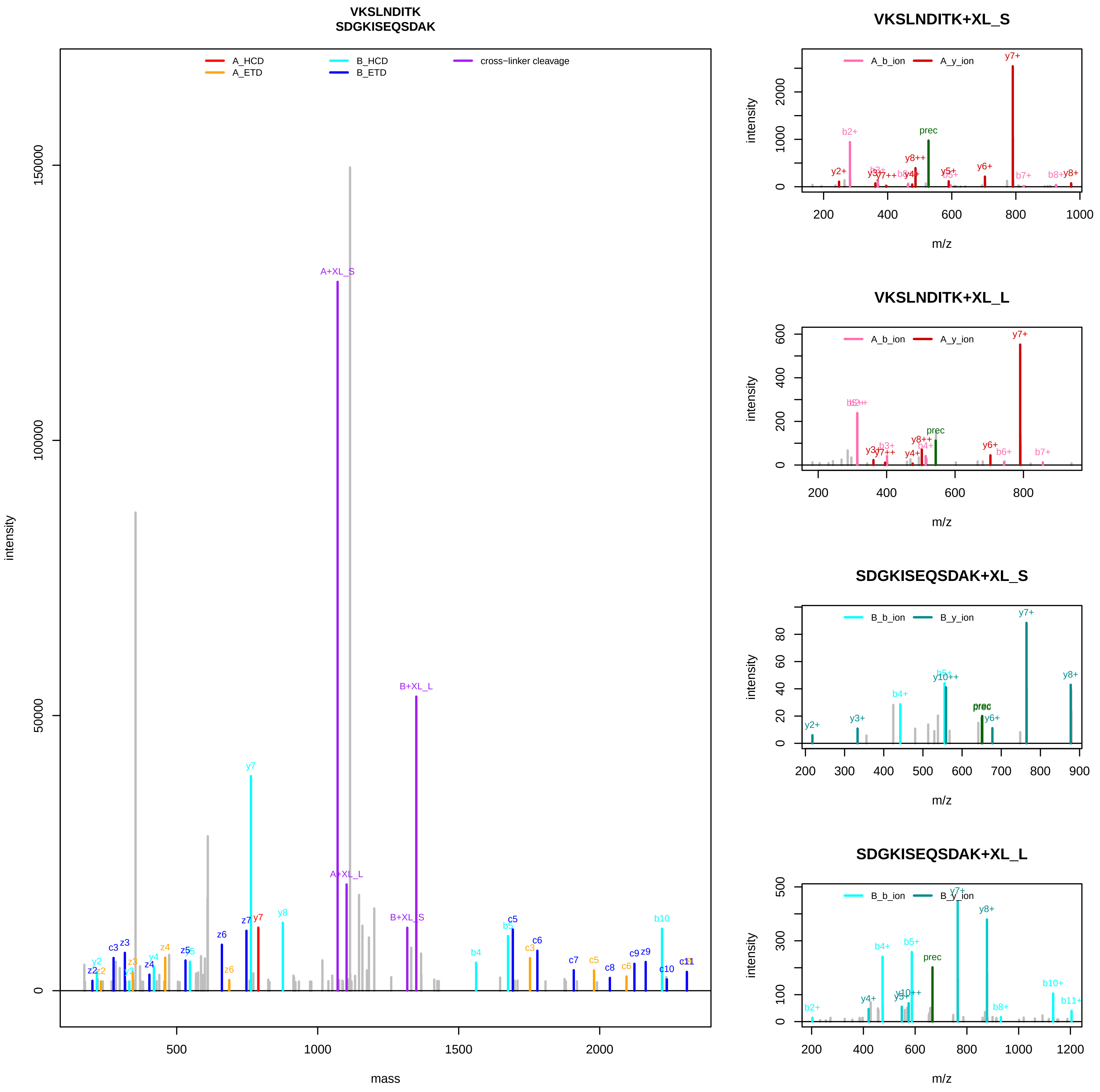

VKSLNDITK
SDGKISEQSDAK
A_HCD
A_ETD
B_HCD
B_ETD
cross−linker cleavage
intensity
mass
0
50000
100000
150000
500
1000
1500
2000
A+XL_S
B+XL_L
A+XL_L
B+XL_S
y7
y8
z7
y7
z6
c5
b5
b10
c6
c3
z3
c3
z4
z5
y5
b4
c7
c5
c8
c6
c9
z9
c10
c11
z2
y2
z2
z3
y3
y4
z4
z6
VKSLNDITK+XL_S
A_b_ion
A_y_ion
intensity
m/z
y7+
b2+
prec
y8++
y2+
y6+
y5+
y8+
b8+
b7+
VKSLNDITK+XL_L
A_b_ion
A_y_ion
intensity
m/z
y7+
prec
y8++
y6+
b6+
b7+
y4+
b3+
SDGKISEQSDAK+XL_S
B_b_ion
B_y_ion
intensity
m/z
y7+
y10++
b4+
y8+
y2+
y3+
y6+
prec
SDGKISEQSDAK+XL_L
B_b_ion
B_y_ion
intensity
m/z
y7+
y8+
b4+
b5+
prec
y4+
b10+
b11+
b2+
b8+
